# Phosphorylcholine antibodies restrict infarct size and left ventricular remodelling by attenuating the unreperfused post‐ischaemic inflammatory response

**DOI:** 10.1111/jcmm.16662

**Published:** 2021-06-30

**Authors:** Niek J. Pluijmert, Rob C. M. de Jong, Margreet R. de Vries, Knut Pettersson, Douwe E. Atsma, J. Wouter Jukema, Paul H. A. Quax

**Affiliations:** ^1^ Department of Cardiology Leiden University Medical Center Leiden The Netherlands; ^2^ Department of Surgery Leiden University Medical Center Leiden The Netherlands; ^3^ Einthoven Laboratory for Experimental Vascular Medicine Leiden University Medical Center Leiden The Netherlands; ^4^ Athera Biotechnologies Stockholm Sweden

**Keywords:** infarct size, inflammatory response, left ventricular remodelling, leukocytes, monocytes, myocardial infarction, phosphorylcholine antibodies

## Abstract

Phosphorylcholine is a pro‐inflammatory epitope exposed on apoptotic cells, and phosphorylcholine monoclonal immunoglobulin (Ig)G antibodies (PC‐mAb) have anti‐inflammatory properties. In this study, we hypothesize that PC‐mAb treatment reduces adverse cardiac remodelling and infarct size (IS) following unreperfused transmural myocardial infarction (MI). Unreperfused MI was induced by permanent ligation of the left anterior descending (LAD) coronary artery in hypercholesterolaemic APOE*3‐Leiden mice. Three weeks following MI, cardiac magnetic resonance (CMR) imaging showed a reduced LV end‐diastolic volume (EDV) by 21% and IS by 31% upon PC‐mAb treatment as compared to the vehicle control group. In addition, the LV fibrous content was decreased by 27% and LV wall thickness was better preserved by 47% as determined by histological analysis. Two days following MI, CCL2 concentrations, assessed by use of ELISA, were decreased by 81% and circulating monocytes by 64% as assessed by use of FACS analysis. Additionally, local leucocyte infiltration determined by immunohistological analysis showed a 62% decrease after three weeks. In conclusion, the local and systemic inflammatory responses are limited by PC‐mAb treatment resulting in restricted adverse cardiac remodelling and IS following unreperfused MI. This indicates that PC‐mAb holds promise as a therapeutic agent following MI limiting adverse cardiac remodelling.

## 1 INTRODUCTION

Therapeutic opportunities to treat patients suffering from an acute myocardial infarction (MI) have improved dramatically with the advent of primary percutaneous coronary interventions^1^ or coronary artery bypass grafting.^2^ However worldwide, immediate revascularization is not possible in a significant portion of the patients suffering from chronic coronary artery disease,^3^ due to anatomical limitations, clinical complications or simply because of unavailable facilities to provide relevant care. Especially in the less developed countries this may be a serious issue. Besides aiming for timely reperfusion and additional therapies to salvage myocardium, intervening in unreperfused transmural MI to modulate cardiac remodelling therefore remains of importance; hence, focus in this study is on unreperfused MI. Transmural MI results in adverse left ventricular (LV) remodelling, characterized by LV dilatation and reduced LV wall thickness, which successively leads to heart failure,^4^ one of the leading causes of death worldwide.^5^

Myocardial infarction triggers a complex inflammatory response, which helps to clear the injured myocardium from dead cardiomyocytes and matrix debris, and ultimately leads to infarct healing and mature scar formation.^6^ However, when the inflammatory response is extended, it may cause viable cardiomyocytes to die.^7^ Necrotic cardiomyocytes release damage‐associated molecular patterns (DAMPs), like high mobility group box‐1 (HMBG1), heat shock protein (HSP), interleukin (IL)‐1α and extracellular RNA (eRNA), which trigger the innate immune system^7^ via Toll‐like receptor (TLR) activation.^8^, ^9^, ^10^ Currently, large randomized controlled trials such as the Canakinumab Antiinflammatory Thrombosis Outcome Study (CANTOS)^11^ and Colchicine Cardiovascular Outcomes (COLCOT)^12^ trials reported promising therapeutic potential of anti‐inflammatory therapies in decreasing cardiovascular events after MI. Additionally, the role of apoptotic cells seems to be more complicated. Uptake of apoptotic cells by macrophages might have anti‐inflammatory effects^13^; on the other hand, it has been suggested that apoptotic cells are immunogenic and pro‐inflammatory.^14^ In addition, effective efferocytosis of apoptotic cardiomyocytes was found to improve the resolution of inflammation after MI.^15^ However, the main part of apoptotic cells in the healing injured myocardium are non‐cardiomyocytes. For instance, apoptotic neutrophils represent a large part of the apoptotic cells in the healing injured myocardium, and their role in inflammation resolution is yet unknown.^7^

Following MI, the production of reactive oxygen species by circulating phagocytes, endothelial cells and cardiomyocytes is increased as a result of the ischaemic event.^16^ These reactive oxygen species are responsible for generating oxidative damage and producing oxidation‐specific epitopes on apoptotic cells, which can act as DAMPs and are recognized by innate immunity.^17^ Phosphorylcholine (PC), the polar headgroup of oxidized phospholipids (oxPLs), is an important oxidation‐specific epitope, present on apoptotic cells but absent on viable cells.^14^ Moreover, phosphorylcholine is present on oxidized LDL (oxLDL), a key player in atherogenesis because of its pro‐inflammatory properties.^18^ It has been shown in mice that a specific clone of IgM autoantibodies against phosphorylcholine, termed E06 or T15 antibodies,^19^ can inhibit the uptake of both apoptotic cells and oxLDL by macrophages in vitro^20^, ^21^ and in vivo^22^ and has anti‐inflammatory properties.^14^ However, if complete cascade systems are present, E06 appears to augment efferocytosis.^23^, ^24^, ^25^ Furthermore, B‐1a and B‐1b cells showed to produce oxidation‐specific epitope‐specific IgM antibodies, which protect against atherosclerosis,^26^, ^27^, ^28^ and it has been found that splenic B cells display an oxidation‐specific epitope‐associated atheroprotective effect, which is initiated through sterile inflammation.^29^ Moreover, low levels of natural IgM phosphorylcholine antibodies are associated with an increased risk of cardiovascular events^30^, ^31^, ^32^, ^33^, ^34^ and resulted in a worsened prognosis regarding patients with an acute coronary syndrome.^35^ In addition, both active and passive immunization with antibodies against phosphorylcholine ameliorates development of atherosclerosis and is proven to be atheroprotective.^36^, ^37^, ^38^ Altogether, these data indicate that blocking phosphorylcholine might be an interesting therapeutic approach to treat cardiovascular disease. However, compared to IgG antibodies, IgM antibodies are not optimal for therapeutic use, because of rapid elimination from plasma, and being unstable, difficult to produce and relatively expensive in addition.

We previously developed a fully human IgG1 directed against human phosphorylcholine (PC‐mAb) and with anti‐inflammatory properties. PC‐mAb blocks oxLDL uptake by macrophages and inhibits vascular remodelling in a mouse model for accelerated atherosclerosis and preserves coronary flow reserve and attenuates atherosclerotic inflammation.^40^ Above all, it attenuates the immediate inflammatory response following myocardial ischaemia‐reperfusion injury in hypercholesterolaemic APOE*3‐Leiden mice, preserving cardiac function with an increased ejection fraction of 33%.^41^ As unreperfused transmural MI yet remains a significant determinant in worldwide morbidity and mortality, pressing heavily on the healthcare system and costs, additional therapeutic effects of PC‐mAb following unreperfused MI might be of interest. Furthermore, hypercholesterolaemia causes a pro‐inflammatory phenotype characterized by monocytosis,^42^, ^43^ making it an important factor to consider in experimental studies. Therefore, in the current study, the effect of PC‐mAb treatment on cardiac function, LV remodelling and the inflammatory response is investigated in hypercholesterolaemic APOE*3‐Leiden mice after initiating unreperfused MI.

## 2 MATERIALS AND METHODS

### 2.1 Animals and diets

All animal experiments were approved by the Institutional Committee for Animal Welfare of the Leiden University Medical Center (LUMC) and conformed to the guidelines from Directive 2010/63/EU of the European Parliament on protection of animals used for scientific purposes. Transgenic female APOE*3‐Leiden mice^44^ aged 8‐10 weeks at the start of a dietary run‐in period were used for this experiment. Mice were fed a semisynthetic Western‐type diet supplemented with 0.4% cholesterol (AB Diets) four weeks prior to surgery, which was continued throughout the complete experiment. Female APOE*3‐Leiden mice were used because of their higher and stable plasma cholesterol and triglyceride levels, confined to the VLDL/LDL‐sized lipoprotein fraction,^45^ and development of advanced aortic atherosclerotic lesions resembling their human counterparts.^46^ Mice were housed under standard conditions in conventional cages and received food and water ad libitum. Plasma levels of total cholesterol and triglycerides were determined for randomization one week before surgery. After 4 hours of fasting, plasma was collected via tail vein bleeding (~50 μL) and examined for total cholesterol and triglyceride levels using commercially available enzymatic kits according to the manufacturer's protocols (11489232 and 11488872, respectively; Roche Diagnostics).

### 2.2 Surgical myocardial infarction model and PC‐mAb treatment

Myocardial infarction was induced by ligation of the LAD coronary artery at day 0 in 12‐ to 14‐week‐old female APOE*3‐Leiden mice as described previously.^47^ Briefly, mice were pre‐anaesthetized with a gas mixture of 5% isoflurane and oxygen and placed in a supine position on a heating pad (37°C). After endotracheal intubation and ventilation (rate 160 breaths/min, stroke volume 190 μL; Harvard Apparatus), mice were kept anaesthetized with 1.5%‐2% isoflurane. Subsequently, a left thoracotomy was performed in the 4th intercostal space and the LAD coronary artery was permanently ligated using a 7‐0 prolene suture. Subsequently, the thorax was closed in layers with 5‐0 prolene suture and mice were allowed to recover. Analgesia was obtained with buprenorphine s.c. (0.1 mg/kg) pre‐operative and 12 hours post‐operative. After surgery, animals were randomly grouped to receive intraperitoneal administration of 10 mg/kg PC‐mAb (known as ATH3G10; Athera Biotechnologies)^39^ every 3rd day or NaCl 0.9% w/v (vehicle) as a control. Sham‐operated animals were operated similarly but without ligation of the LAD and received injections with NaCl 0.9% w/v (sham).

After 2 days or 3 weeks, under general anaesthesia with 1.5%‐2% isoflurane, mice were euthanized by bleeding and explantation of the heart. Hearts were immersion‐fixated for 24 hours in 4% paraformaldehyde and embedded in paraffin. Blood samples were collected and used for serum analysis. The heart and bodyweight were measured from all animals using a digital scale.

### 2.3 Cardiac magnetic resonance imaging

Left ventricular dimensions, function and IS were assessed 2 days and 3 weeks after surgery by using 7‐Tesla CMR imaging (Bruker Biospin) to obtain contrast‐enhanced and cine CMR images. Mice were pre‐anaesthetized with 5% isoflurane in a gas mixture of oxygen and kept anaesthetized with 1.5%‐2% isoflurane. Respiratory rate was monitored by a respiration detection cushion, which was placed underneath the thorax and connected to a gating module to monitor respiratory rate (SA Instruments, Stony Brook). Image reconstruction was performed using Bruker ParaVision 5.1 software.

#### 2.3.1 Infarct size

Infarct size was determined with contrast‐enhanced CMR imaging after injection of 150 μL (0.5 mmol.mL) of gadolinium‐DPTA (Gd‐DPTA, Dotarem, Guerbet) via the tail vein. To acquire a set of 14 contiguous 0.7 mm contrast‐enhanced slices in short‐axis orientation, a gradient echo sequence (FLASH) was used. Imaging parameters were as follows: echo time of 1.9 ms, repetition time of 84.16 ms, field of view of 33 mm^2^ and a matrix size of 192 × 256.

#### 2.3.2 Left ventricular function

Left ventricular function was assessed with a high‐resolution 2D FLASH cine sequence to acquire a set of 9 contiguous 1 mm slices in short‐axis orientation covering the entire heart. Imaging parameters were as follows: echo time of 1.49 ms, repetition time of 5.16 ms, field of view of 26 mm^2^ and a matrix size of 144 × 192.

#### 2.3.3 Image analysis

MR Analytical Software System (MASS) for mice (MEDIS) was used for image analysis. LV endo‐ and epicardial borders were delineated manually, and a reference point was positioned by an investigator blinded to treatment. End‐diastolic and end‐systolic phases and the contrast‐enhanced areas were identified automatically, and the percentage of infarcted myocardium, LV end‐diastolic volume (EDV), LV end‐systolic volume (ESV) and LV ejection fraction (EF) were computed.

### 2.4 LV fibrous content and LV wall thickness

Paraffin‐embedded hearts were cut into serial transverse sections of 5 µm along the entire long axis of the LV, and every 50th section was stained with Sirius Red. Collagen deposition was used as an indicator of the fibrotic area, and LV fibrous content was determined by planimetric measurements of all sections and calculated as fibrotic area divided by the total LV wall surface area.

Left ventricular wall thickness was analysed in five different sections centralized in the infarct area. Per section wall thickness was measured at three places in the infarct area, both border zones, and at two places in the intraventricular septum. All measurements were performed using the ImageJ 1.47v software program (NIH).

### 2.5 Local inflammatory response

For analysis of the cardiac inflammatory response, a subpopulation was selected, and sections were stained using antibodies against leucocytes (anti‐CD45, 550539; BD Pharmingen). The number of leucocytes was expressed as a number per 0.25 mm^2^ in the septum (2 areas), border zones (2 areas) and infarcted myocardium (3 areas).

### 2.6 FACS analysis

To examine the effect of PC‐mAb therapy on the acute inflammatory response, mice were killed and blood samples were collected after 2 days. To study the systemic effects, whole blood was analysed for monocytosis. Total circulating leucocytes were determined using a semi‐automatic haematology analyser F‐820 (Sysmex Corporation).

For FACS analysis, 35 μL of whole blood was incubated for 30 minutes on ice with directly conjugated antibodies directed against Ly6C‐FITC (AbD Serotec), Ly6G‐PE (BD Pharmingen), CD11b‐APC (BD Pharmingen) and CD115‐PerCP (R&D Systems). Monocytes were gated based on their expression profile: CD11b‐positive, Ly6G‐negative and CD115‐positive. Data were analysed using FlowJo software (Tree Star Inc).

### 2.7 CCL2 and PC‐mAb ELISA

A PC‐mAb ELISA kit (Athera Biotechnologies) was used to determine serum PC‐mAb concentrations, with a secondary antibody detecting human IgG. To study the effects of PC‐mAb on systemic inflammation, inflammatory cytokine concentration of chemokine (C‐C motif) ligand 2 (CCL2) was determined using an ELISA kit (Cat. No. 555260, BD Biosciences).

### 2.8 Phosphorylcholine and TLR4 co‐localization

The presence of Toll‐like receptor 4 (TLR4) and phosphorylcholine co‐localization in the infarct area was investigated by immunohistochemistry. TLR4 was stained using specific antibodies against TLR4 (anti‐CD284, AHP1822, Bio‐Rad Laboratories Inc). Phosphorylcholine was stained using the same antibody (Athera Biotechnologies) as was used for treatment.

### 2.9 Statistical analysis

Values were expressed as mean ± SEM. Comparisons of parameters between the sham, PC‐mAb and vehicle groups were made using 1‐ or 2‐way analysis of variance (ANOVA) with repeated measures and Tukey's post hoc correction for multiple pairwise comparisons. Comparisons were made between PC‐mAb and vehicle using unpaired Student's *t* tests. A value of *P* < .05 was considered a significant difference. Statistical procedures were performed using SPSS 26.0 (IBM Corporation) and GraphPad Prism 8.0 (GraphPad Software).

## 3 RESULTS

### 3.1 Animal characteristics

No differences in bodyweight (BW) were observed between the PC‐mAb group (20.4 ± 0.3 g) compared to vehicle (20.5 ± 0.4 g) and sham (19.6 ± 0.3 g). Possible cardiac hypertrophy was assessed by determining heart weight (HW) and heart‐to‐bodyweight (HW‐BW) ratio. PC‐mAb treatment reduced both HW (134 ± 5 mg) and HW‐BW ratio (6.6 ± 0.3) compared to vehicle (HW: 167 ± 9 mg, *P* = .008; HW‐BW ratio: 8.2 ± 0.4, *P* = .009; Table 1). In addition, baseline total plasma cholesterol (TC) levels were equally distributed; however, PC‐mAb therapy following unreperfused MI lowered TC levels after 3 weeks compared to vehicle (11.1 ± 0.6 vs 15.8 ± 1.1 mmol/L, *P* = .008) as well as its internal T0 control (11.1 ± 0.6 vs 15.0 ± 1.4 mmol/L, *P* = .013; Table 1).

**TABLE 1:** Plasma lipid levels and animal characteristics

	T (wk)	sham	MI	MI
			vehicle	PC‐mAb
		N = 13	N = 16	N = 14
TC (mmol/L)	0	15.5 ± 1.5	13.7 ± 1.0	15.0 ± 1.4
	3	13.1 ± 1.0	15.8 ± 1.1	11.1 ± 0.6^a^, ^b^
TG (mmol/L)	0	2.4 ± 0.2	2.9 ± 0.2	3.0 ± 0.2
	3	2.4 ± 0.2	1.9 ± 0.2^c^	1.6 ± 0.1^c^, ^d^
BW (g)	0	20.7 ± 0.5	20.9 ± 0.5	21.1 ± 0.3
	3	19.6 ± 0.3	20.5 ± 0.4	20.4 ± 0.3
HW (mg)	3	144 ± 8	167 ± 9	134 ± 5^a^
HW/BW ratio (mg/g)	3	7.3 ± 0.3	8.2 ± 0.4	6.6 ± 0.3^a^

Note

Values are mean ± SEM.

Abbreviations: BW, bodyweight; HW, heart weight; TC, plasma total cholesterol; TG, triglycerides.

^a^ p < .01 vs vehicle.

^b^ p < .05 vs T0.

^c^ p < .001 vs T0.

^d^ p < .05 vs sham.

### 3.2 PC‐mAb concentrations, cellular mechanisms and phosphorylcholine‐TLR4 co‐localization

To confirm the observed effects to be the result of PC‐mAb treatment, circulating PC‐mAb serum concentrations were determined using ELISA. PC‐mAb levels were detectable only in the PC‐mAb group after 2 days (32 ± 8 µg/ml) and 3 weeks (36 ± 6 µg/ml), confirming the absence of a native immune response against PC‐mAb, and were not observed in the vehicle or sham‐operated groups (Figure S1). In addition, it was shown before that PC‐mAb binds to late apoptotic cells with strong affinity.^39^, ^41^ Moreover, treatment with oxidized low‐density lipoprotein of cultured peripheral blood mononuclear cells isolated from human blood showed suppressed CCL2 levels following concomitant PC‐mAb treatment (Figure S2).

**FIGURE 1:**
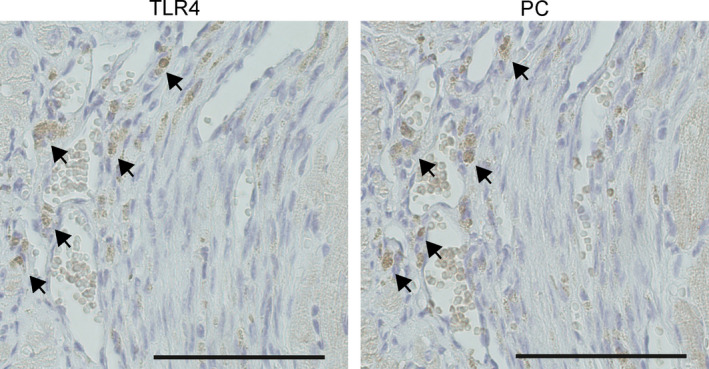
Phosphorylcholine and TLR4 co‐localization. Representative images of the infarct area 2 d following unreperfused MI with co‐localized TLR4 (A) and phosphorylcholine (B) staining. Scale bar = 50 μm

As the rationale for using PC‐mAb to treat adverse cardiac remodelling following unreperfused MI was to inhibit the pro‐inflammatory response, TLR4, as a triggering factor of the inflammatory response,^8^ and phosphorylcholine co‐localization was investigated. As can be appreciated from Figure 1, TLR4 is indeed localized at comparable areas in the infarct area as is phosphorylcholine.

### 3.3 PC‐mAb reduces contrast‐enhanced CMR assessed LV infarct size

**FIGURE 2:**
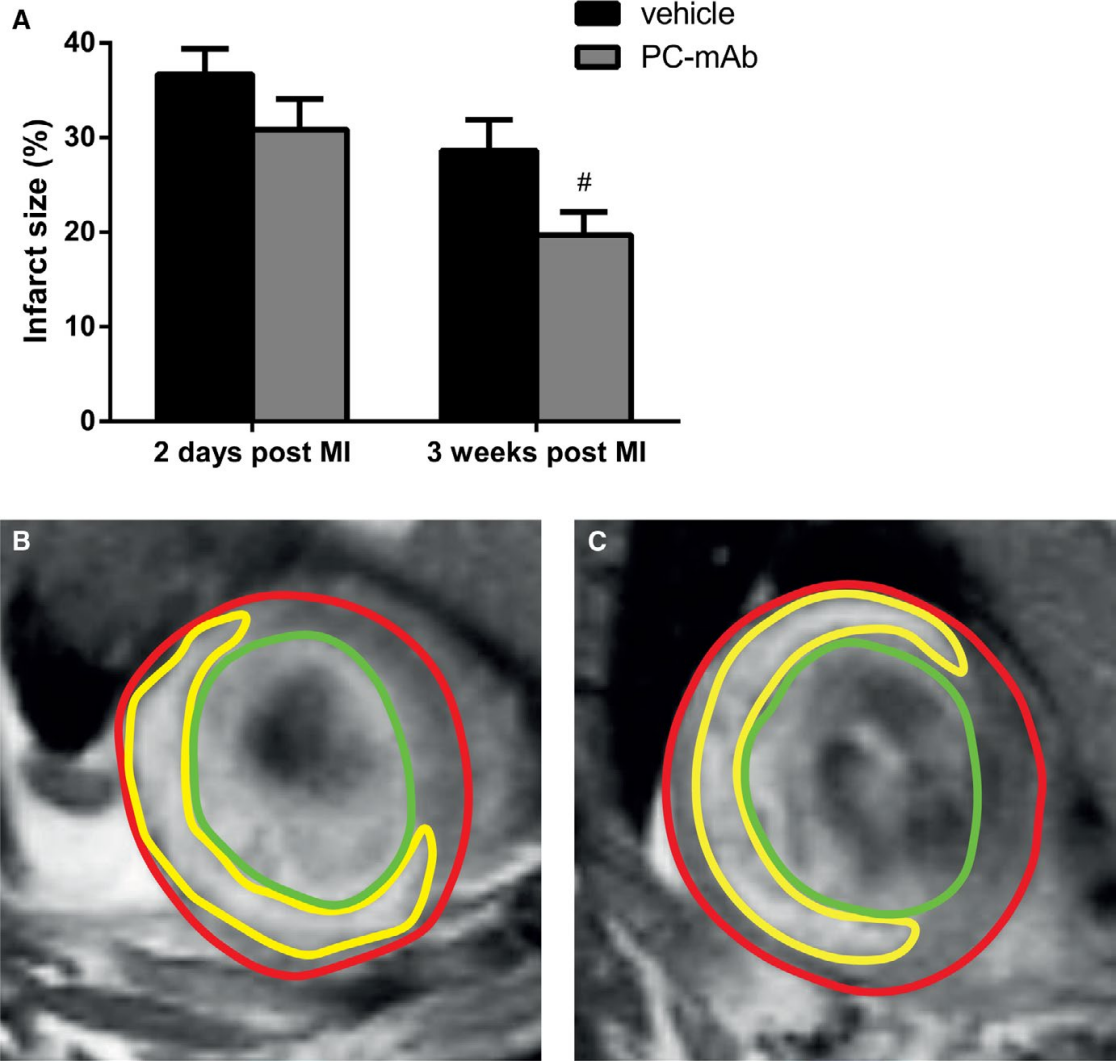
Quantification of infarct size using contrast‐enhanced CMR imaging. Infarct size is quantified as percentage of the LV mass (A; n = 14‐16 per group). Representative contrast‐enhanced CMR images 2 d following unreperfused MI after vehicle (B) and PC‐mAb (C) treatment. Epicardial borders are indicated by red lines, endocardial borders by green lines and infarct area by yellow lines. Data are mean ± SEM. ^#^ *P* < .05 vs vehicle

Baseline IS was assessed using contrast‐enhanced CMR two days following unreperfused MI. No differences in IS could be observed between the PC‐mAb‐treated group and the vehicle group at baseline (30.9 ± 3.2% vs 36.7 ± 2.7%, *P* = .175). However, after 3 weeks, PC‐mAb treatment compared to vehicle treatment showed a smaller IS (19.7 ± 2.4% vs 28.6 ± 3.3%, *P* = .042; Figure 2A). Interestingly, IS following unreperfused MI was significantly smaller after 3 weeks compared to 2 days in both vehicle and PC‐mAb group. This may indicate that after initial transitory infarct oedema as may occur after 2 days, some degree of favourable infarct remodelling occurs as observed after 3 weeks.

### 3.4 PC‐mAb reduces LV dilatation but does not affect LV function

**FIGURE 3:**
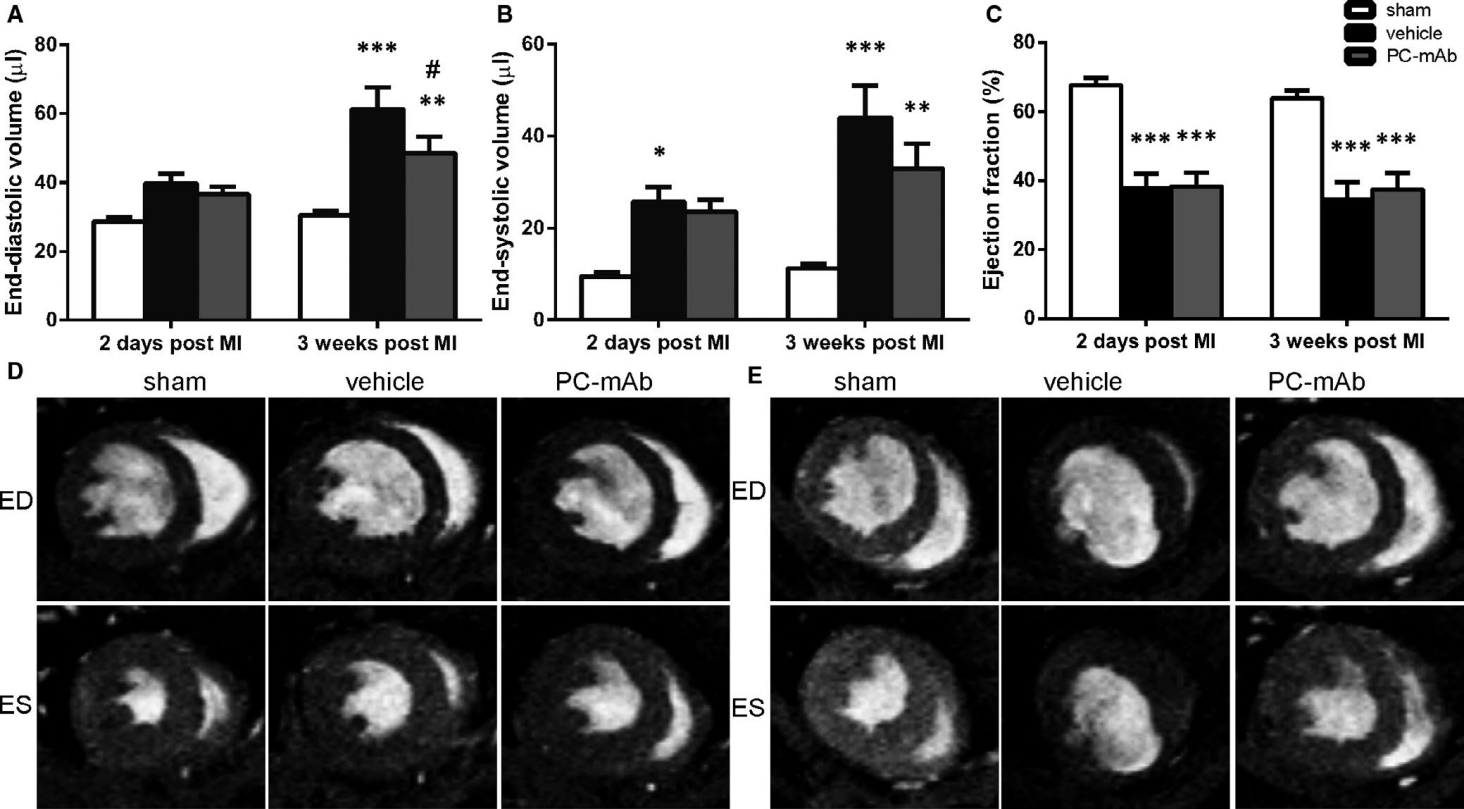
CMR imaging for quantification of LV volumes and function. LV volumes, EDV (A) and ESV (B), and function, EF (C), were assessed 2 d and 3 wk following unreperfused MI in hypercholesterolaemic APOE*3‐Leiden mice (n = 12‐16 per group). Representative CMR images of transversal short‐axis views at end‐diastole (ED) and end‐systole (ES) 2 d (D) and 3 wk (E) after MI in the sham, vehicle and PC‐mAb groups. Data are mean ± SEM. ^#^ *P* < .05 vs vehicle, *P < .05, **P < .01, ***P < .001 all vs sham

To investigate the effect of PC‐mAb treatment on LV dilatation, serial cine CMR images were made 2 days and 3 weeks following unreperfused MI. After 2 days, no differences could be observed between vehicle and PC‐mAb treatment regarding EDV (39.7 ± 2.8 μL vs 36.7 ± 2.2 μL; Figure 3A) and ESV (25.7 ± 3.1 μL vs 23.5 ± 2.7 μL; Figure 3B). After 3 weeks, ESV differed between the PC‐mAb and vehicle group (32.9 ± 5.5 μL vs 44.0 ± 7.0 μL, *P* = .163; Figure 3B), which was reduced even more pronouncedly in terms of EDV (48.6 ± 4.7 μL vs 61.3 ± 6.3 μL, *P* = .048; Figure 3A), indicating restricted LV dilatation. No differences in EF were observed in the PC‐mAb group as compared to vehicle treatment both 2 days (38.3% ± 4.0% vs 37.8% ± 4.1%; Figure 3C) and 3 weeks after unreperfused MI (37.4% ± 4.7% vs 34.4% ± 5.1%; Figure 3C).

### 3.5 PC‐mAb reduces LV fibrous content and ameliorates wall thickness

**FIGURE 4:**
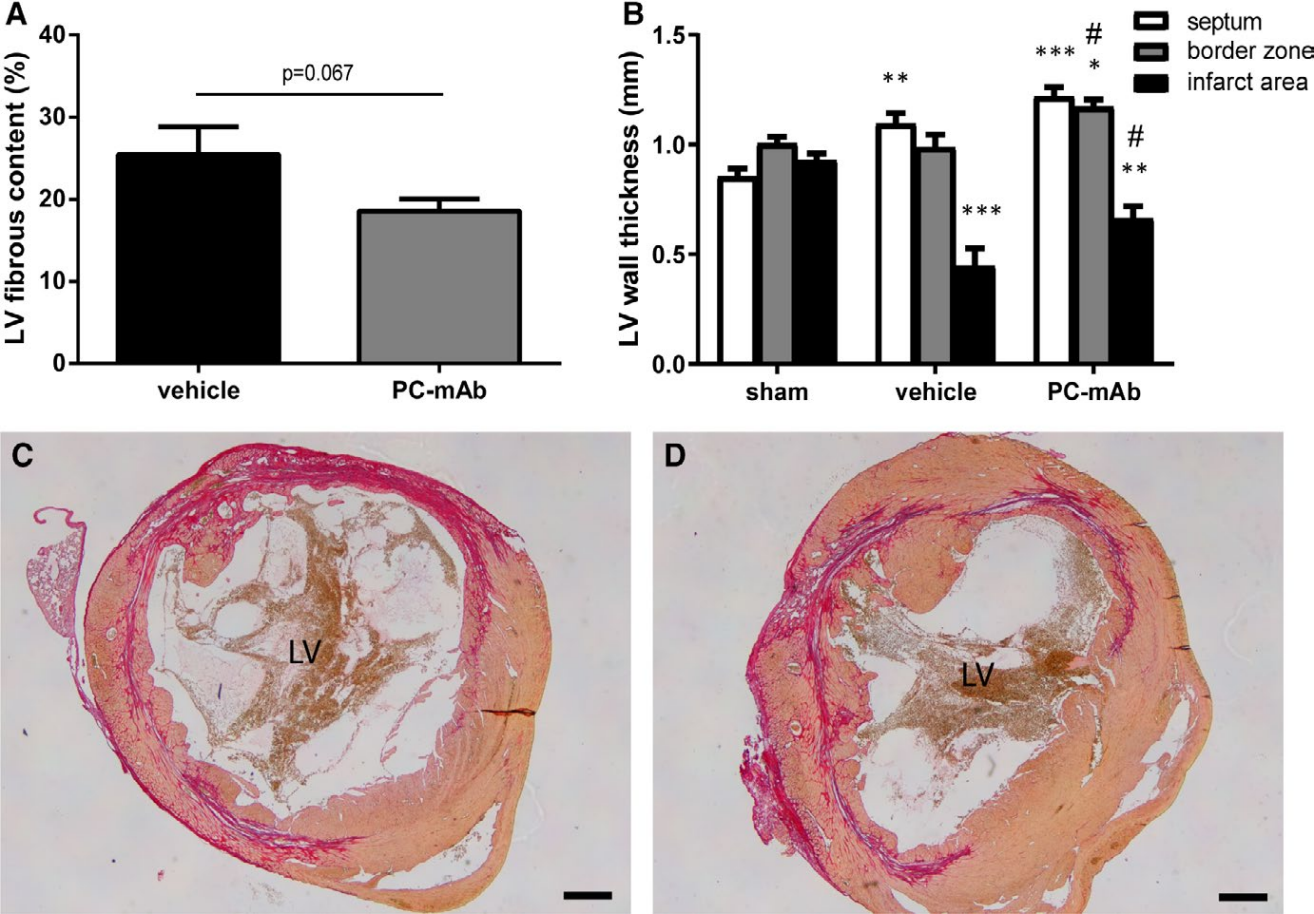
Histological analysis of LV fibrous content and wall thickness 3 wk after MI. LV fibrous content (A) was determined as the area of the LV occupied by collagen with Sirius red staining (n = 9‐10 per group). LV wall thickness (B) was measured in 3 areas specified as the interventricular septum, border zone and infarct area. Representative Sirius Red–stained images after vehicle (C) or PC‐mAb (D) treatment. Scale bar = 500 μm. Data are mean ± SEM. ^#^ *P* < .05 vs vehicle, *P < .05, **P < .01, ***P < .001 all vs sham

Three weeks following unreperfused MI, PC‐mAb treatment showed a strong trend towards a decreased LV fibrous content compared to vehicle treatment (18.6% ± 1.4% vs 25.5% ± 3.4%, *P* = .067; Figure 4A), confirming our results obtained with contrast‐enhanced CMR imaging. In addition, LV wall thickness was increased in the septum of both PC‐mAb (1.22 ± 0.04 mm, *P* < .001) and vehicle group (1.10 ± 0.05 mm, *P* = .001) compared to sham (0.85 ± 0.04 mm; Figure 4B), indicating compensatory cardiac hypertrophy suggesting viable myocardium to compensate for the infarcted myocardium. Moreover, LV wall thickness was increased in the border zone and infarct area in the PC‐mAb group compared to the vehicle group (border zones: 1.17 ± 0.03 mm vs 0.99 ± 0.06 mm, *P* = .012; infarct area: 0.66 ± 0.06 mm vs 0.45 ± 0.08 mm, *P* = .035; Figure 4B). Both the increased LV wall thickness in the border zone, a likely result of augmented cardiac hypertrophy, as the preserved LV wall thickness in the infarct area seemed to be a result of the PC‐mAb treatment indicating improved cardiac remodelling.

### 3.6 PC‐mAb attenuates the systemic inflammatory response

**FIGURE 5:**
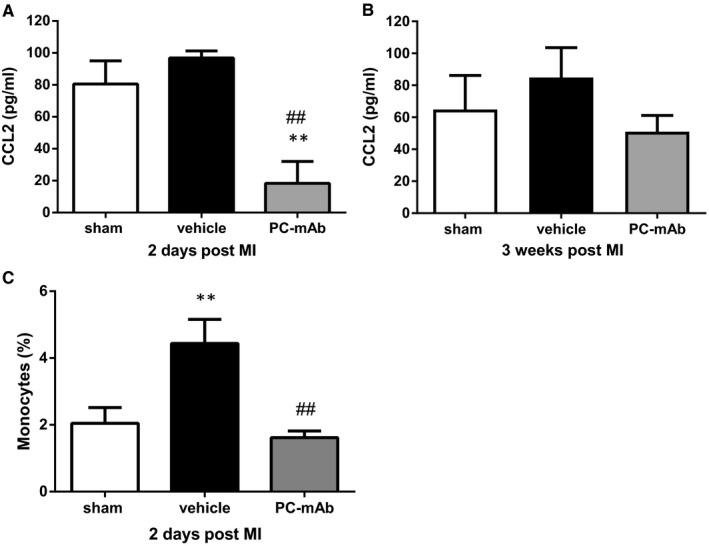
Analysis of the immediate and late systemic inflammatory response. Serum levels of CCL2 were determined using ELISA as a measure of systemic inflammation, 2 d (A; n = 6‐8 per group) and 3 wk (B; n = 9‐10 per group) following unreperfused MI in hypercholesterolaemic APOE*3‐Leiden mice. Circulating monocytes were determined after 2 d using FACS analysis and expressed as percentage of total leucocytes (C; n = 6‐8 per group). Data are mean ± SEM. ^##^ *P* < .01 vs vehicle, **P < .01 vs sham

After 2 days, CCL2 concentrations were significantly reduced in the PC‐mAb group (18.3 ± 13.7 pg/mL) compared to both the sham (80.5 ± 14.5 pg/mL, *P* = .006) and vehicle group (96.8 ± 4.4 pg/mL, *P* = .002; Figure 5A), suggesting PC‐mAb to reduce the systemic inflammatory response. However, after 3 weeks, no differences could be observed in serum CCL2 concentrations between all groups suggesting a transient effect on the immediate systemic inflammatory process (Figure 5B).

Furthermore, the effect of PC‐mAb treatment 2 days after unreperfused MI regarding circulating monocytes was investigated using FACS analysis. Circulating monocytes expressed as the percentage of total leucocytes were decreased in the PC‐mAb group compared to the vehicle group (1.6% ± 0.2% of 1.3*10^5^ leucocytes vs 4.4% ± 0.7% of 1.0*10^5^ leucocytes, *P* = .003; Figure 5C). Moreover, the percentage of circulating monocytes was comparable to the sham group (2.0% ± 0.5% of 1.2*10^5^ leucocytes), demonstrating PC‐mAb treatment attenuates monocytosis.

### 3.7 PC‐mAb limits the local inflammatory response

**FIGURE 6:**
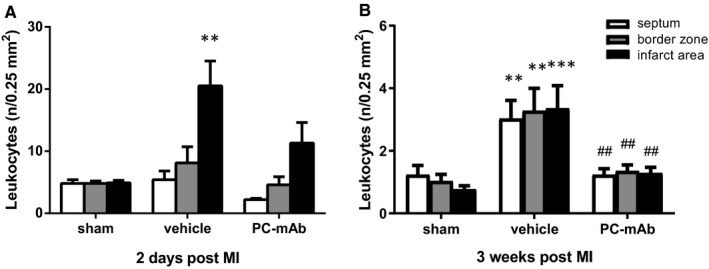
Quantification of the immediate and late local inflammatory response. As a measure of local inflammation, the number of CD45 positive cells (leucocytes) was counted per specific area (interventricular septum, border zone and infarct area). Bars represent the average number of leucocytes per field of view in the specific areas following unreperfused MI after 2 d (A; n = 4‐5 per group) and 3 wk (B; n = 9‐10 per group). Data are mean ± SEM. ^##^ *P* < .01 vs vehicle, **P < .01, ***P < .001 both vs sham

In addition, a striking decrease in local leucocyte infiltration in all areas was observed in the PC‐mAb‐treated group compared to the vehicle group 3 weeks after unreperfused MI (septum: 1.2 ± 0.2 vs 3.0 ± 0.6 per 0.25 mm^2^, *P* = .008; border zones: 1.4 ± 0.2 vs 3.3 ± 0.7 per 0.25 mm^2^, *P* = .009; infarct area: 1.3 ± 0.2 vs 3.4 ± 0.7 per 0.25 mm^2^, *P* = .004; Figure 6B), suggesting PC‐mAb treatment to reduce the local inflammatory response in case of unreperfused MI. Moreover, numbers of leucocytes in the PC‐mAb group were comparable with the sham group (septum: 1.2 ± 0.3, border zones: 1.0 ± 0.2, infarct area: 0.8 ± 0.1 per 0.25 mm^2^), suggesting, to some degree, an accelerated and better resolution of the inflammatory response.

As the acute phase of the inflammatory response is crucial following MI, the number of leucocytes that infiltrated the cardiac tissue 2 days after unreperfused MI was investigated. In the infarct area, an increased number of leucocytes was observed in the vehicle group compared to the sham group (20.5 ± 4.0 vs 4.9 ± 0.4 per 0.25 mm^2^, *P* = .007), while no difference was observed between the PC‐mAb group and the sham group (11.3 ± 3.3 vs 4.9 ± 0.4 per 0.25 mm^2^, *P* = .337; Figure 6A), suggesting that PC‐mAb treatment dampened the acute local inflammatory response.

## 4 DISCUSSION

PC‐mAb is a human phosphorylcholine monoclonal IgG1 antibody with anti‐inflammatory properties.^39^ In our study, post‐ischaemic administration of PC‐mAb attenuated the immediate systemic inflammatory response after 2 days and the late local inflammatory response after 3 weeks. As a result, IS and LV dilatation were restricted with concomitant preservation of LV wall thickness. Adverse left ventricular remodelling is one of the mechanisms responsible for development of heart failure,^4^ known for its high morbidity and mortality rates worldwide,^5^ especially in the absence of reperfusion. PC‐mAb therapy compared to control limited adverse cardiac remodelling as observed by restricted LV dilatation. Additionally, PC‐mAb treatment compared to control reduced HW and HW/BW ratio, indicating reduced compensatory cardiac hypertrophy, which is another hallmark of adverse cardiac remodelling and heart failure.^48^ Moreover, PC‐mAb treatment compared to control causes a decreased IS, which has been directly linked to heart failure and mortality following MI.^49^ These results suggest PC‐mAb treatment to be a potential therapeutic agent against ischaemic‐induced heart failure in the absence of reperfusion. Previously, we demonstrated PC‐mAb treatment to additionally preserve cardiac function following myocardial ischaemia‐reperfusion injury,^41^ suggesting a point of no return regarding preservation of cardiac function in the absence of reperfusion, endorsing the relevance and impact of selecting different models of myocardial ischaemia.^50^

Inflammation plays an important role following MI, being responsible for removing necrotic and apoptotic cells, thereby improving infarct healing and mature scar formation.^6^ However, extensive inflammation may cause death of viable cardiomyocytes and enhances adverse LV remodelling.^7^ As PC‐mAb treatment compared to control reduces the local inflammatory response following unreperfused MI after 3 weeks but not after 2 days, as indicated by reduced leucocyte infiltration, it is suggested that PC‐mAb therapy restricts the deleterious extensive late inflammatory response limiting adverse LV remodelling,^7^ while interestingly the beneficial immediate local inflammatory response is not inhibited.

Although context dependent, it has been shown that oxPLs are agonists for TLR4 signalling resulting in generation of chemokines and cytokines like CCL2, interleukin (IL‐)6 and IL‐8.^51^, ^52^ TLR4 and phosphorylcholine are co‐localized in the infarct area following unreperfused MI, suggesting phosphorylcholine to be a ligand for TLR4 signalling in the infarcted myocardium, endorsing PC‐mAb as a potential post‐ischaemic therapy. By expressing anti‐inflammatory properties, PC‐mAb seems to reduce LV remodelling as shown by decreased IS and LV dilatation as compared to control, while preserving LV wall thickness. Following MI, cardiomyocytes partially become apoptotic,^6^ and apoptotic cells express oxidized lipids on their outer membrane,^53^ which are immunogenic.^14^ It has been demonstrated that natural and monoclonal E06/T15 antibodies against phosphorylcholine bind to apoptotic cells,^20^, ^53^ inhibiting the inflammatory response.^14^ Therefore, the observed reduced IS is suggested to be the result of a suppressed inflammatory response associated with enhanced efferocytosis^23^, ^24^, ^25^ and activation of reparative cells sparing viable cardiomyocytes.^54^ The preserved LV wall thickness probably is a result of the dampened inflammatory response, although it cannot be excluded that it partially is a direct result of the decreased LV dilatation and limited adverse cardiac remodelling.^55^

CCL2 is a chemoattractant known for its ability to attract inflammatory leucocytes to sites of tissue injury,^56^ for example after myocardial ischaemia‐reperfusion injury.^57^ Although these attracted leucocytes promote removal of dead tissue and infarct healing, it has been shown that CCL2‐deficient mice show decreased recruitment of macrophages into the infarcted myocardium that coincides with decreased LV remodelling following myocardial ischaemia‐reperfusion injury.^57^ This agrees with our finding of decreased CCL2 serum concentration and restricted adverse LV remodelling upon PC‐mAb treatment. Previously, PC‐mAb was shown to reduce CCL2 levels produced by human monocytes stimulated with oxLDL in vitro and regarding accelerated atherosclerosis local expression of CCL2 in the vessel wall was inhibited.^39^ Furthermore, it is known that blood CCL2 levels are increased in hypercholesterolaemic APOE*3‐Leiden mice.^58^ Therefore, PC‐mAb treatment is suggested to reduce the systemic inflammatory response by binding phosphorylcholine on apoptotic cells and/or oxLDL, which contributes to the restricted adverse LV remodelling as a result of reduced serum CCL2 concentrations.

Hypercholesterolaemia causes a pro‐inflammatory phenotype, which is characterized by monocytosis,^42^ mainly caused by an increase in the pro‐inflammatory Ly‐6C^hi^ subset. It has been shown that following unreperfused MI in hypercholesterolaemic APOE^‐/‐^ mice, more Ly‐6C^hi^ monocytes are recruited into the infarct area, which resulted in decreased LV function^59^ and impaired infarct healing.^60^ Additionally, myocardial ischaemia‐reperfusion injury in hypercholesterolaemic APOE*3‐Leiden mice preceded by a pre‐ischaemic Ly‐6C^hi^ monocytosis resulted in a decreased LV function as well, but was paradoxically coinciding with a reduced IS.^43^ This underscores the complex interplay between different mechanisms of ischaemia and concomitant luxating cardiovascular risk factors and the necessity of selecting appropriate experimental models to investigate hypotheses correctly.^50^ Following unreperfused MI, we showed that PC‐mAb therapy compared to control reduces circulating monocytes, accompanied by a reduced IS and restricted LV dilatation.

In conclusion, PC‐mAb treatment following unreperfused transmural MI limits adverse cardiac remodelling and IS as compared to control, likely by ameliorating the immediate inflammatory response upon myocardial ischaemia. Interestingly, PC‐mAb seems to mitigate both the atherosclerotic as the ischaemic inflammatory process related to MI. Until now, phase 1 studies showed good safety and tolerability and an additional phase 2a, randomized, placebo‐controlled, double‐blind, multicentre pilot study is currently running in patients suffering an acute MI. Therefore, PC‐mAb treatment might be a potential valuable novel therapeutic strategy to restrict inflammation and adverse cardiac remodelling, improving outcome in ischaemic heart disease when immediate reperfusion is unavailable or not possible.

## CONFLICTS OF INTEREST

KP is a named inventor on patent and minor shareholder in Athera Biotechnologies. The remaining authors have nothing to disclose.

## AUTHOR CONTRIBUTION

**Niek J. Pluijmert:** Conceptualization (supporting); Data curation (equal); Formal analysis (equal); Investigation (lead); Visualization (lead); Writing‐original draft (lead). **Rob CM de Jong:** Data curation (equal); Formal analysis (equal); Investigation (supporting); Visualization (supporting); Writing‐original draft (supporting). **Margreet R de Vries:** Conceptualization (supporting); Formal analysis (supporting); Validation (supporting); Writing‐review & editing (supporting). **Knut Pettersson:** Conceptualization (supporting); Formal analysis (supporting); Methodology (supporting); Validation (supporting); Writing‐review & editing (supporting). **Douwe E Atsma:** Methodology (supporting); Supervision (supporting); Validation (supporting); Writing‐review & editing (supporting). **J Wouter Jukema:** Conceptualization (supporting); Methodology (supporting); Validation (supporting); Writing‐review & editing (supporting). **Paul HA Quax:** Conceptualization (lead); Formal analysis (supporting); Methodology (lead); Supervision (lead); Validation (lead); Writing‐review & editing (lead).

## Supporting information

Figure S1‐S2

## Data Availability

The data sets generated and/or analysed during the current study are available from the corresponding author upon reasonable request.
