# Supplementary material for: Phosphorylcholine antibodies restrict infarct size and left ventricular remodelling by attenuating the unreperfused post‐ischaemic inflammatory response
Source: J Cell Mol Med. 2021 Jun 30;25(16):7772–82. doi: 10.1111/jcmm.16662 (PMC8358891; doi:10.1111/jcmm.16662)
Supplement: Supplementary file 1 — Figure S1‐S2 [file JCMM-25-7772-s001.pdf]

Supplementary Information

Phosphorylcholine antibodies restrict infarct size and left ventricular remodeling by attenuating the unreperfused post-ischemic inflammatory response in hypercholesterolemic APOE*3-Leiden mice

Niek J. Pluijmert[1], Rob C. M. de Jong[2,3], Margreet R. de Vries[2,3], Knut Pettersson[4], Douwe E. Atsma[1], J. Wouter Jukema[1,3], Paul H. A. Quax[2,3]

[1] Department of Cardiology, Leiden University Medical Center, Leiden, The Netherlands
[2] Department of Surgery, Leiden University Medical Center, Leiden, The Netherlands
[3] Einthoven Laboratory for Experimental Vascular Medicine, Leiden University Medical Center, Leiden, The Netherlands
[4] Athera Biotechnologies, Stockholm, Sweden

Supplementary Figures

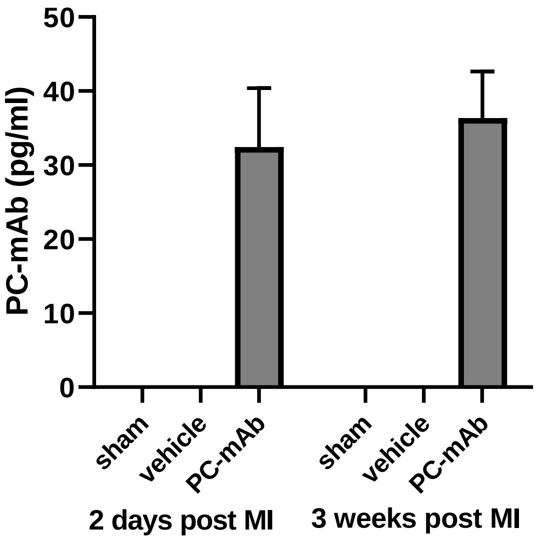

Supplemental Figure 1: Circulating serum PC-mAb concentrations determined by ELISA 2 days and 3 weeks after MI. PC-mAb levels were only detectable in the PC-mAb treated group.

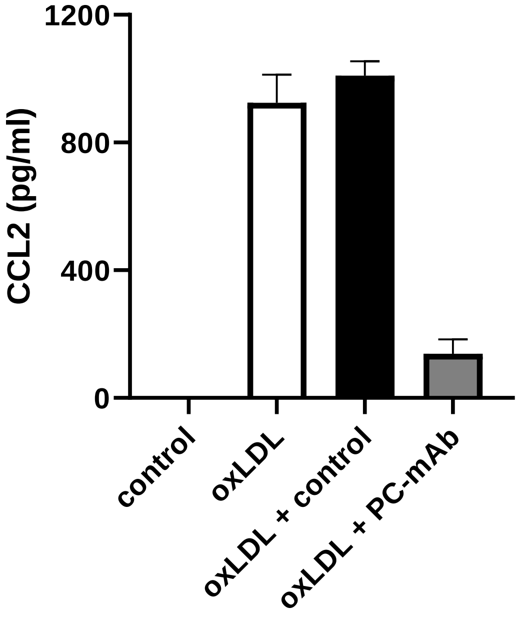

Supplemental Figure 2: PC-mAb affecting expression levels of CCL2. Cultured PBMCs isolated from human blood were treated with oxLDL in the presence or absence of PC-mAb with IgG isotype as a control. PC-mAb treatment resulted in a clear suppression of CCL2 levels.
